# Prevention of the transmission of *Babesia rossi* by *Haemaphysalis elliptica* in dogs treated with Nexgard^®^

**DOI:** 10.1051/parasite/2019051

**Published:** 2019-08-21

**Authors:** Frederic Beugnet, Wilfried Lebon, Christa de Vos

**Affiliations:** 1 Boehringer-Ingelheim 29 Av Tony Garnier 69007 Lyon France; 2 ClinVet PO Box 11186 Universitas 9321 Bloemfontein South Africa

**Keywords:** Afoxolaner, NexGard, Dog, *Haemaphysalis elliptica*, *Babesia rossi*, Prevention

## Abstract

This experimental study aimed to determine the efficacy of Afoxolaner (NexGard^®^) to prevent *Babesia rossi* transmission by *Haemaphysalis elliptica* ticks on dogs. The study included three groups of seven dogs each. Groups 1 and 2 remained untreated, whereas group 3 dogs received NexGard^®^ on Day 0. All dogs were infested by 50 *Haemaphysalis elliptica* adult ticks: Group 1 on Day 2, Group 2 on Day 28 and Group 3 on Days 2 and 28. The ticks were originally nymphs having fed on *B. rossi* infected donor dogs. Their infection rate, assessed by PCR, was 12.8% at Day 2 and 6% at Day 28. On Days 0, 7, 14, 21, 28, 35, 42, 49 and 56, and in case of suspicion of babesiosis, blood samples were collected for blood smears, PCR and ELISA. The *B. rossi* infection rate in the untreated group 1 was 100% (6/6, as one dog was inadvertently treated on Day 15 and removed from statistical analysis). The infection rate was 57.1% (4/7) in group 2, and 0% (0/7) in the afoxolaner treated group 3 at all time-points until the end of the study on Day 56. After tick removal and count 144 h after each infestation, the control groups had an arithmetic mean of ticks of 23.8 (group 1) and 26.8 (group 2). No tick was recovered from any treated dogs. This study demonstrated that NexGard^®^ protected dogs against infection by *B. rossi* for at least 28 days.

## Abstract (fr)

Cette étude expérimentale visait à déterminer l’efficacité de l’Afoxolaner (NexGard^®^) dans la prévention de la transmission de *Babesia rossi* par les tiques *Haemaphysalis elliptica* chez les chiens. L’étude comprenait 3 groupes de 7 chiens chacun. Les groupes 1 et 2 sont restés non traités alors que les chiens du groupe 3 ont reçu NexGard^®^ au jour 0. Tous les chiens ont été infestés par 50 tiques adultes *Haemaphysalis elliptica* : le groupe 1 le jour 2, le groupe 2 le jour 28 et le groupe 3 les jours 2 et 28. Les tiques étaient à l’origine des larves s’étant nourries sur des chiens infectés par *B. rossi*. Leur taux d’infection, évalué par PCR, était de 12,8 % au jour 2 et de 6 % au jour 28. Aux jours 0, 7, 14, 21, 28, 35, 42, 49 et 56, et en cas de suspicion de babésiose, des échantillons de sang ont été prélevés pour des frottis sanguins, PCR et ELISA. Le taux d’infection par *B. rossi* dans le groupe 1 non traité était de 100 % (6/6, un chien ayant été traité par inadvertance le jour 15 et retiré de l’analyse statistique). Il était de 57,1 % (4/7) dans le groupe 2. Le taux d’infection par *B. rossi* dans le groupe 3 traité par l’Afoxolaner était de 0 % (0/7) à tous les temps jusqu’à la fin de l’étude au jour 56. Après retrait des tiques et dénombrement 144 heures après chaque infestation, les groupes de contrôle présentaient une moyenne arithmétique de tiques de 23,8 (groupe 1) et de 26,8 (groupe 2). Aucune tique n’a été retrouvée chez les chiens traités. Cette étude a démontré que NexGard^®^ protégeait les chiens contre l’infection par *B. rossi* pendant au moins 28 jours.

## Introduction

Ticks are endemic throughout the world with more than 800 species, of varying biology and geographic distribution [2]. Ticks commonly infest dogs and are vectors of various canine vector-borne pathogens including *Babesia, Ehrlichia, Anaplasma*, and *Borrelia* [1, 4]*.* Canine babesiosis is a clinically significant tick-borne protozoan disease [2]. Piroplasms in dogs are divided into two morphologically distinct groups, the large forms and the small forms (i.e. > or < the radius of erythrocytes). The large forms have been classified into three separate species, *Babesia canis, B. rossi* and *B. vogeli* [4, 20]*.* Each of these *Babesia* species is transmitted by a different tick vector, i.e. *Dermacentor reticulatus* for *B. canis*, *Rhipicephalus sanguineus* for *B. vogeli* and *Haemaphysalis elliptica* for *B. rossi*. The clinical signs of canine babesiosis vary from a mild transient illness to acute disease. *Babesia vogeli* is considered to be the least pathogenic species, inducing mild clinical signs like anaemia and fever, whereas *B. canis* often induces acute babesiosis including strong febrile syndrome, anaemia, haemoglobinuria, splenomegaly, and icterus. *Babesia rossi* is considered the most pathogenic, inducing severe haemolysis that rapidly results in death, with possible neurological signs related to intravascular thromboembolism, especially in brain blood vessels [2, 4, 11, 15, 20].

*Babesia rossi* is considered the primary agent of canine babesiosis, also called “biliary fever”, in South Africa, with jackals as reservoir hosts [5, 21, 22]. It often induces very severe forms, including a typical neuro-babesiosis that clinically resembles human *Plasmodium falciparum* neuro-malaria [5, 11, 21]. Clinical complications – including cerebral effects, intestinal haemorrhage, haemoglobinuria, acute renal failure and/or pulmonary oedema – are considered common by South African veterinarians and death can occur in more than 10% of cases [11, 15]. *Babesia* infection follows transmission of the causative agent to a dog during attachment and feeding by an infected tick. It can take more than 24 h of attachment to transmit the parasite from tick to dog, which provides a window of opportunity to kill the tick and prevent transmission [6, 14, 19]. The incubation period between the tick bite and the onset of clinical signs can be as short as two weeks, and signs are the result of intravascular red blood cell destruction following intra-erythrocytic asexual parasite reproduction [11, 15]. Clinically infected dogs are usually diagnosed through the observation of sporozoites in erythrocytes through a stained blood smear. Veterinarians treat canine babesiosis in South Africa with antiparasitic drug administration (i.e. imidocarb dipropionate) as well as blood transfusions and supportive care [11, 15].

Published studies demonstrating the utility of tick control compounds on dogs have been focused on their acaricidal efficacy against a broad range of ixodid tick species [10, 20]. Some experimental studies have shown the protective effect provided by topical (spot on and collar formulations) and oral acaricides against *Babesia* transmission [3, 8, 9, 12, 13, 18, 25]. Efficacy is based on the repellent/irritant effect by contact, inhibition of attachment and blood meal, and/or a quick speed of kill. The protective effect of an acaricidal molecule given orally to dogs and acting systemically is less obvious as the ticks must attach and start to feed before being killed [3, 9, 25]. Nevertheless, it has been demonstrated that pathogens need some time to be transmitted [14, 19]. Moreover, on top of the killing effect, an acaricide may also block the feeding physiology of the tick. Afoxolaner, fluralaner and sarolaner, from the isoxazoline class, have been demonstrated to reduce the risk of transmission of the European *Babesia canis* species by *Dermacentor reticulatus* ticks [3, 9, 24]. In this article, we present the results of an experimental study to assess the efficacy of afoxolaner*,* administered orally in a soft chewable formulation (Nexgard^®^, Boehringer-Ingelheim), against *Haemaphysalis elliptica* ticks infected with *Babesia rossi* in dogs.

## Materials and methods

The study was a parallel group, randomised, negative controlled, efficacy study [16]. It included three groups of seven dogs each at the start of the study, selected from an enrolled group of 24 dogs.

Group 1 dogs (*n* = 7) received no product (negative control) and were infested with *B. rossi* infected ticks on Day 2. Group 2 dogs (*n* = 7) were also negative controls, and were infested with *B. rossi* infected ticks on Day 28. Group 3 dogs (*n* = 7) were treated orally with NexGard^®^ on Day 0, and were infested with *B. rossi* infected ticks on Days 2 and 28.

The included dogs were clinically healthy as verified by a veterinarian on Day −7 (group 1 and three dogs) or on Day 21 (group 2 dogs); they were seronegative and polymerase chain reaction (PCR−) negative prior to enrolment; they were ≥6 months old (Day −7 or Day 21); they weighed ≥ 10 kg [Day −2 or Day 27]; they were not clinically pregnant; and they had not been treated with a long-acting topical or systemic acaricide/insecticide (including products with repellent activity) during the 12 weeks preceding Day 0.

Sixteen dogs were enrolled on Day −7 and a further eight on Day 21. The 16 dogs enrolled on Day −7 were weighed on Day −2 and the 14 heavier dogs were allocated to groups 1 and 3. The 14 dogs included in the study on Day −2 were ranked within sex in descending order of individual body weights and subsequently blocked into seven blocks of two dogs each. Dog weight range was 11.0–26.5 kg and they were 13–88 months old.

The additional eight dogs enrolled on Day 21 were weighed on Day 27 and the seven heavier dogs were allocated to group 2. Veterinary examinations were performed on Day −7 and on all dogs included in the study on Days 7, 14, 21, 28, 35, 42, 49 and 56. Animals were monitored daily for any health changes, and if any such case occurred, a clinical examination was performed.

The dog cages were located in an indoor animal unit, with environmentally controlled temperature (20 °C ± 4 °C). The animals were kept individually in cages (3.0 × 2.1 m). However, animals still had visual and auditory contact with conspecifics.

Each cage was fitted with a sleeping bench. At least one toy/chew was made available to each dog (replenished weekly). The protocol and accommodation were approved by ethics committees in consideration of the South African National Standard SANS 10386: “The care and use of animals for scientific purposes.”

The dogs were fed once a day. Water bowls were replenished at least twice daily.

NexGard^®^ (afoxolaner 2.27% w/w) was administered to group 3 dogs on Day 0. Weighing between 11 and 22 kg, all treated dogs received a 68 mg afoxolaner chew as per the label (one chew for dogs from 10 to 25 kg). The chew was administered by placement in the back of the oral cavity over the tongue to initiate swallowing. No chew was lost due to spitting or vomiting by any of the animals; therefore, no re-dosing was necessary.

Dogs were infested with 50 adult (25 males and 25 females) *H. elliptica* ticks from a batch infected with *B. rossi* on Day 2 (groups 1, 3) and Day 28 (groups 2, 3). To facilitate tick infestation, ticks were released on the dog, manually restrained in an infestation crate for 10 min. Ticks were released onto the dorsal areas, behind the shoulder blades of the dog. Before each infestation, a sample of ticks was taken from the batch, and the infectivity was confirmed by PCR analyses. These ticks were fed at the nymphal stages on *B. rossi* infected dogs, using a method previously developed for *Babesia canis* [13]. They were then allowed to moult into the adult stage in order to obtain an infected batch of adult ticks.

The Babesia rossi isolate is maintained at ClinVet and was originally collected from the blood of one dog presenting clinical signs of severe babesiosis in a veterinary clinic in Bloemfontein (South Africa) in October 2017.

The ticks used were from a laboratory-bred colony of *H. elliptica*. The tick colony originated from >20 engorged female ticks collected on one dog in Bloemfontein in February 2014.

Ticks were counted by *in situ* thumb counting on Day 4 (groups 1, 3) and on Day 30 (groups 2, 3) (i.e. 48 h after infestation). Removal tick counts were conducted on Day 8 (groups 1, 3) and on Day 34 (groups 2, 3) (i.e. 144 h after infestation). The collected ticks were classified as live/dead and free/attached.

Blood samples (3 mL in EDTA tubes) were collected for PCR and immunofluorescence assay (IFA) analysis prior to study start, on Days 2 (prior to tick infestation), 14, 21, 28 (prior to tick infestation), 42, 49 and 56. Blood samples were also collected at any times when high body temperature (>39.4 °C) and/or clinical signs suggestive of babesiosis were observed on dogs (i.e. anaemia, diarrhoea, neurological signs, sleepiness, lethargy, inappetence, reddish urine) [11, 15, 21].

All dogs suspected of developing babesiosis were examined and two blood smears were prepared and evaluated for the presence of *B. rossi* sporozoites [11, 15, 21]. If a dog was positive for *B. rossi* on a blood smear, blood was kept for PCR analysis, and the dog immediately received rescue treatment. Rescue treatment consisted of 1 mL/20 kg body weight Berenil^®^ RTU (diminazine aceturate) IM, followed by 1.2 mL/20 kg body weight Forray 65^®^ (imidocarb dipropionate) SC the day following a dose of Berenil^®^. This therapy has been demonstrated to clear infections [11, 15].

IFAs were performed at ARC-Onderstepoort Veterinary Institute/Research (ARC-OVR, http://www.arc.agric.za/arc-ovi/Pages/ARC-OVI-Homepage.aspx).

Rectal body temperatures were recorded for all included dogs three times a week from Days 7 to 28 (groups 1, 3) and Days 33 to 56 (groups 2, 3). The completion of the animal phase of the study was on Day 28 for the animals in group 1 and on Day 56 for the dogs in groups 2 and 3.

For PCR, total genomic DNA was isolated from whole blood samples, using the GeneJet genomic DNA isolation kit (Thermo Scientific). PCR entailed the use of primers Babesia2F (5′–GGAAGGAGAAGTCGTAACAAGGTTTCC–3′) and Brossi2R (5′–CTCAGAACTTCAGGCCATCCAAAG–3′) specific for the amplification of the 18S–ITS1–5.8S–ITS2 region of *B. rossi* rDNA [20]. Up to 400 ng isolated DNA served as a template for PCR amplification of the target region. PCR entailed the use of Phire Green Hot Start II PCR Master Mix (Thermo Scientific) in a 20 μL reaction volume containing 500 nM of each primer. Thermal cycling entailed 98 °C for 5 min followed by 45 cycles of 98 °C for 5 s, 68 °C for 5 s, and 72 °C for 30 s, followed by a final elongation at 72 °C for 1 min. PCR products were analysed using agarose gel electrophoresis and results were documented. A PCR product of approximately 567 bp indicated the presence of the *B. rossi* rDNA target region in the sample. Positive, negative, no template, as well as internal amplification controls were included in each run. The PCR setup and conditions used in this study resulted in a minimum detection sensitivity of ≥5 target copies per PCR, and no cross-reaction with *B. canis* and *B. vogeli* DNA could be detected (data not shown).

For serology, the blood was allowed to clot at room temperature for 15 min prior to being centrifuged. For serum separation, the blood samples were centrifuged at room temperature. The serum was transferred to two pre-labelled cryo tubes (primary and duplicate aliquots) and stored at −18 °C or below after collection until shipment to the serology laboratory. For detection of *B. rossi* antibodies, the sera were diluted and results were expressed as 1/80 or 1/40 (indicating the sample being positive when fluorescence at dilution 1/80 or 1/40 was observed) or negative (no fluorescence).

The primary efficacy criterion was the number of dogs that became infected with *B. rossi*. A dog had a viable infection with *B. rossi* if the dog was positive for *B. rossi* on blood smear; or tested serologically positive for *B. rossi* antibodies; or tested positive for *B. rossi* by PCR analysis.

The percentage of transmission blocking efficacy for the treated groups was calculated as follows:

$$\mathrm{Transmission}\;\mathrm{blocking}\;\mathrm{efficacy}\;(\%)=100\times (T_{c}-T_{t})/T_{c},$$

where:

*T*_c_ = total number of untreated control dogs (group 1 or 2) that became infected;
*T*_t_ = total number of dogs that became infected in the IVP group (group 3).

In consideration of available guidelines, it was decided that the acaricidal efficacy calculations should be based on arithmetic mean values rather than geometric mean values.

Efficacy against ticks was calculated for the NexGard treated group at the assessment day according to the following formula:

$$\mathrm{Acaricidal}\;\mathrm{efficacy}\;(\%)=100\times (M_{c}-M_{t})/M_{c},$$

where:

*M*_c_ = Arithmetic mean number of live ticks (categories 1 and 2) on dogs in the negative control groups (group 1 or 2) at a specific time point;
*M*_t_ = Arithmetic mean number of live ticks (categories 1 and 2) on dogs in the IVP group (group 3) at a specific time point.

The statistical unit was the individual dog. The groups were compared using an ANOVA (Proc. GLM procedure in SAS) with a treatment effect on both untransformed and logarithmic transformed tick (count + 1) data. SAS Version 9.3 TS Level 1M2 was used for all the statistical analyses. The level of significance of the formal tests was set at 5%, all tests were two-sided.

## Results

Fever and clinical signs associated with *B. rossi* infection were observed in 10 of the control dogs (6/6 dogs in control group 1, and 4/7 dogs in control group 2) with positive blood smears, confirming the pathogenicity of this *Babesia* species (Tables 1 and 4) [11, 15, 21]. Symptoms appeared 10–14 days after tick infestation. No infections could be observed on blood smears prepared for dogs treated with NexGard^®^ (group 3) for the duration of the study.

**Table 1:** Signs reported during the daily health observations.

Group	Animal ID	Day	Clinical sign(s)	Concomitant therapy required
1	287 8A5	5	Vomited in cage (resolved on Day 6)	No
		14	Fever, Comatose, laboured breathing (died on Day 14)	Yes
	5B3 B85	15	Fever, Blood in faeces (resolved on Day 16)	Yes
	86A B0D	14	Fever, Lethargy	Yes
	86D 365	14	Fever, Lethargy	Yes
	885 EB2	14	Fever, Lethargy	Yes
	DF7 C86	15	Fever, Lethargy	Yes
	DF6 793	14	Inappetance (resolved on Day 15)	No
		24	Vomited in cage (resolved on Day 25)	No
		47	Dark urine (resolved on Day 48)	No
2	5A5 E30	39	Fever, Lethargy	Yes
	5A6 66D	39	Fever, Lethargy	Yes
	5A6 66D	39	Fever, Lethargy	Yes
	5A6 66D	39	Fever, Lethargy	Yes
3	698 163	53	Vomited (resolved on Day 54)	No

Dog DF6 793 (group 1) was inadvertently rescue treated on Day 15 (without a positive diagnosis on Day 14) and was excluded from all statistical analyses.

On Day 14, dog 287 8A5 (group control 1) was found collapsed in the cage, with increased and laboured breathing. A blood smear confirmed *B. rossi* infection with secondary acute respiratory distress syndrome. The rescue treatment was initiated immediately, including specific treatment and fluid therapy; nevertheless, the dog’s condition did not improve (Table 2). The dog died approximately two hours following the onset of treatment. A full *post-mortem* examination was performed and samples for histopathological analysis were collected. The *post-mortem* indicated congested lungs, liver and kidneys with widespread petechial haemorrhages, cyanosis and watery blood. *B. rossi* was also confirmed positive through PCR.

**Table 2:** Diagnosis and prescription of concomitant medication.

Group	Animal ID	Day of diagnosis	Diagnosis	Drug name and prescription
1	287 8A5; 5B3 B85; 86A B0D; 86D 365; 885 EB2	14	*Babesia rossi*	Berenil RTU: 0.05 mL/kg SC once-off; Forray 65: 0.06 mL/kg SC once-off (day following Berenil); Corticoid injection 0.08 mL/kg SC id for two days
	DF6 793*; DF7 C86	15		
2	5A5 E30; 5A6 66D; 5A6 66D; 5A6 66D	39		
1	287 8A5	14	*Babesia rossi*	Ringer lactate 1L IV; Whole blood transfusion: ±400 mL IV

* Dog DF6 793 (group 1) was inadvertently rescue treated on Day 15 (without a positive diagnosis on Day 14) and was excluded from all statistical analyses.

Group 1 – Negative control dogs infested with ticks on Day 2.

Group 2 – Negative control dogs infested with ticks on Day 28.

Dog DF6 793 (group 1) was inadvertently rescue treated on Day 15 due to fever and suspicion of babesiosis, which was finally not confirmed, and was therefore excluded from all statistical analyses.

Abnormal signs observed during daily observations are summarised in Table 1. Most signs observed were associated with infection with *B. rossi.*

The arithmetic mean number of attached *H. elliptica* ticks counted at 48 h and 144 h after infestation on Day 2 (groups 1, 3) and Day 28 (groups 2, 3) is shown in Table 3. The untreated control groups had an arithmetic mean ranging from 20.3 to 26.8 on these assessment days, demonstrating successful tick challenges.

**Table 3:** Acaricidal efficacy using arithmetic mean for *Haemaphysalis elliptica* ticks.

	Control group 1	Control group 2	Afoxolaner treated group 3		
Day	Mean	Mean	Mean	Percentage efficacy	ANOVA *p*-value
Day 4	23.8	/	0.0	100	<0.0001
Day 8	26.8	/	0.0	100	<0.0001
Day 30	/	21.4	0.0	100	<0.0001
Day 34	/	20.3	0.0	100	<0.0001

Based on arithmetic means, statistically significantly (*p* < 0.05) less ticks were observed on the NexGard^®^ treated group compared to the untreated control groups (groups 1 and 2) on all assessment days. NexGard^®^ provided 100% efficacy against *H. elliptica* ticks on all assessment days.

The infection rates of tick batches with *B. rossi* used for challenge on Days 2 and 28 were 12.8% and 6%, respectively.

The IFA results recorded during this study for each dog are summarised in Table 4. *Babesia rossi* antibodies were detected in all dogs (6/6) in the untreated control group 1, four dogs (4/7) in the untreated control group 2, and no dogs (0/7) in the afoxolaner treated group 3.

**Table 4:** Summary of body temperatures, blood smear analysis, IFA and PCR performed during the study.

Group	Animal ID	Body temperatures			Blood smear positive (Day)	PCR positive (Day)	IFA positive (Day)
		Minimum (°C)	Maximum (°C)	Study day when temperature > 39.4 °C			
1	86D 365	38.0	40.1	14	14	14	21, 28
	5B3 B85	37.4	40.6	14	14	14	21, 28
	885 EB2	38.2	40.4	14	14	14	21, 28
	287 8A5	38.4	38.8	–	14	14	–
	DF7 C86	38.2	39.6	28	15	14	21, 28
	86A B0D	37.5	40.5	7, 9, 14, 21, 26, 28	14	14	21, 28
	DF6 793*	37.7	39.1	(dog removed due to treatment)			
2	698 1B0	37.8	40.4	33	–	–	–
	885 0F5	37.8	39.2	–	–	–	–
	5A6 66D	38.1	39.9	39	39	39	42
	5A5 E30	38.5	40.4	28, 39, 40, 42, 56	39	39	42
	86A B70	38.0	39.6	35	–	–	–
	86A 6A0	37.8	40.9	35, 39, 40	39	39	42
	86A 8C3	39.4	40.6	21, 33, 39, 40, 42, 49, 56	39	39	42
3	698 163	38.3	40.0	21, 28, 35, 37, 40	–	–	–
	284 00D	37.4	38.8	–	–	–	–
	86A D40	38.1	40.5	7, 12, 14, 16, 19, 21, 23, 26, 28, 33, 35, 37, 40, 41	–	–	–
	5A7 D8F	37.8	39.2	–	–	–	–
	86A A21	37.8	39.1	–	–	–	–
	DF7 CEB	38.4	39.8	40	–	–	–
	B2C 926	37.4	38.7	–	–	–	–

Dog DF6 793 (group 1) was rescue treated on Day 15 (without a positive diagnosis confirmed) and was excluded from all statistical analyses.

–: negative/not detected at all assessments.

The PCR results recorded during this study for each dog are summarised in Table 4. *Babesia rossi* DNA was detected in all dogs (6/6) dogs in the untreated control group 1, four dogs (4/7) in the untreated control group 2, and no dogs (0/7) in the afoxolaner treated group 3.

A dog was regarded as positively infected if it was positive for *B. rossi* on blood smear, tested serologically positive for *B. rossi* antibodies, or tested positive for *B. rossi* by PCR analysis. In this experimental study, all dogs infected with *B. rossi* tested positive for all three diagnostic techniques (blood smear, PCR and IFA). Based on the criteria above, 100% (6/6) dogs were infected in control group 1, 57.1% (4/7) in control group 2, and 0% (0/7) in NexGard^®^ treated group 3 (for Days 2 and 28).

## Discussion

*Babesia species* are considered to be pathogens with slow transmission by ticks, needing more than 48 h after tick attachment [14, 19]. Mehlhorn et al. have indicated a time of 48–72 h being required after initiation of attachment for sporogony of *B*. *canis* to occur in *D*. *reticulatus*, and for sporozoites to be able to be transmitted by the tick, as observed by microscopy [17]. Piesman & Spielman, 1980 observed transmission of *Babesia microti* in 36 h by *Ixodes dammini* to mice [23]. Consequently, it seems possible to decrease or prevent the transmission of *Babesia* species by using acaricidal treatment on animals. These treatments need to provide enough sustained speed of kill or a repellent effect. Thus, recent studies have been conducted in dogs to assess the preventive effect of anti-tick product against *Babesia* transmission [3, 9, 12, 13, 18, 24]. In all these models, as in the present study, adult ticks were allowed to feed on the animals for at least four days in order for the transmission to be observed in the negative control groups.

To conduct these experimental studies, the generation of a large batch of ticks with an adequate *Babesia* infection rate is necessary. This requires breeding tick colonies and to have *Babesia* infected donor dogs on which tick nymphs are fed [12]. The rate of infection in moulted adult ticks is calculated by conducting PCR on a batch of ticks. Regardless of which sex or if both sexes transmit the *Babesia,* the tick infection rate needs to be sufficient to successfully transmit the infection to the untreated (control) dogs. In this study, the infection rate was 12.8% at Day 2 (estimated from 40 ticks) and 6% (estimated from 50 ticks) at Day 28. This means that on average, 3–6 adult ticks deposited on each dog were infective. As a result, 10/14 untreated dogs were infected and were rescue treated immediately after diagnosis. The rescue treatment was particularly important when dealing with this highly pathogenic agent *Babesia rossi*. Even though the dogs were checked on a daily basis to observe the first sign of infection, i.e. lethargy and fever, one of the dogs died during this experiment. The early rescue treatment based on the appearance of fever for the majority of the infected control dogs in this study can explain the absence of the complete list of symptoms of babesiosis, including fever syndrome, anaemia, haemolysis, haemoglobinuria, and icterus [11, 15]. In line with published studies indicating an average of two weeks of incubation, we observed the first signs in control dogs on Day 14 after tick challenge. In all suspected cases, stained blood smears were positive for intra-erythrocytic forms of *Babesia rossi*. The PCR exam on blood provided the exact same results, and all infected dogs became seropositive within three weeks after tick challenge.

The acaricidal treatment assessed in this experiment was an oral formulation, meaning that ticks need to attach and start to feed before being killed. No data were available in regards to the efficacy of afoxolaner against *Haemaphysalis elliptica*, but only *Dermacentor reticulatus* [3, 7]. This study demonstrated 100% acaricidal efficacy of NexGard^®^ against *Haemaphysalis elliptica* within 48 h (thumb counts) for 28 days. The sustained efficacy made it possible to prevent transmission of *Babesia rossi* in all treated dogs after two infective challenges, on Days 2 and 28, respectively.
